# Essential Genes Embody Increased Mutational Robustness to Compensate for the Lack of Backup Genetic Redundancy

**DOI:** 10.1371/journal.pone.0168444

**Published:** 2016-12-20

**Authors:** Osher Cohen, Matthew Oberhardt, Keren Yizhak, Eytan Ruppin

**Affiliations:** 1 School of Computer Sciences and Sackler School of Medicine, Tel Aviv University, Tel Aviv, Israel; 2 Center for Bioinformatics and Computational Biology, University of Maryland, College Park, MD, United States of America; Johns Hopkins University, UNITED STATES

## Abstract

Genetic robustness is a hallmark of cells, occurring through many mechanisms and at many levels. Essential genes lack the common robustness mechanism of genetic redundancy (i.e., existing alongside other genes with the same function), and thus appear at first glance to leave cells highly vulnerable to genetic or environmental perturbations. Here we explore a hypothesis that cells might protect against essential gene loss through mechanisms that occur at various cellular levels aside from the level of the gene. Using *Escherichia coli* and *Saccharomyces cerevisiae* as models, we find that essential genes are enriched over non-essential genes for properties we call “*coding efficiency”* and “*coding robustness*”, denoting respectively a gene’s efficiency of translation and robustness to non-synonymous mutations. The coding efficiency levels of essential genes are highly positively correlated with their evolutionary conservation levels, suggesting that this feature plays a key role in protecting conserved, evolutionarily important genes. We then extend our hypothesis into the realm of metabolic networks, showing that essential metabolic reactions are encoded by more “robust” genes than non-essential reactions, and that essential metabolites are produced by more reactions than non-essential metabolites. Taken together, these results testify that robustness at the gene-loss level and at the mutation level (and more generally, at two cellular levels that are usually treated separately) are not decoupled, but rather, that cellular vulnerability exposed due to complete gene loss is compensated by increased mutational robustness. Why some genes are backed up primarily against loss and others against mutations still remains an open question.

## Introduction

Robustness is a defining hallmark of all evolved, stable living systems, including single celled organisms. Throughout evolution, different robustness features have evolved within many cellular circuits (e.g., in genetic, protein, and metabolic flux networks). These features operate through a variety of mechanisms [1] to protect cell functions. Most basically, the perturbations that robustness protects against can occur either externally in the environment, or internally through genetic or other intracellular changes. While there are several hypotheses for how genetic robustness evolves [2], it is unclear whether adaptations typically evolve directly to protect against mutations in essential genes, or indirectly as a byproduct of adaptations to changes in the environment. Mutation rates, population size, environmental diversity of a species (specialized or generalist) and metabolic capacities all play a role in building the selective pressure that drives development of robustness, but disentangling the causes from the effects is a standing challenge [3,4].

A common form of robustness is redundancy. It can be expressed either directly, whereby two genes (or other cellular elements, such as enzymes, transcription factors, etc.) exist in a cell for the same function (thus, e.g., one non-essential gene can back up a homologous gene if the second becomes inactivated), or indirectly, when an alternative pathway fulfills the missing role of an inactivated gene. Essential genes, by definition, are not redundant–knocking out any essential gene will kill the cell. Since essential genes are common in cells and play many important roles, a question that naturally arises is if and what secondary mechanisms might exist that provide robustness to essential genes? It has been shown that adaptive (direct) robustness can evolve to compensate for structures with low intrinsic robustness (e.g., which are a byproduct of evolution of some other property) [5]. Is this the case for essential genes? If so, what kind of adaptive mechanisms have evolved? This question becomes especially important when considering bacterial pathogens, where resistance to antibiotics poses a tremendous public health threat. A better understanding of the robustness mechanisms backing up essential genes in such organisms could potentially explain cases of resistance formation when they are targeted, and help to design more effective drugs.

As techniques for probing genomes have matured, essential genes have been catalogued and examined in depth for an increasing number of organisms. Notably, several studies have been done to exhaustively catalog essential genes in *Escherichia coli* for multiple media. Similarly, essential genes have been catalogued for many other bacteria and also eukaryotes, including *Saccharomyces cerevisiae* (yeast). With the screening of essential genes across whole genomes, efforts have turned towards analyzing their features. For example, essential genes have been assessed for evolutionary conservation, protein length distribution, and amino acid usage [6,7]. The experimental methods that enabled essentiality screens are now often complimented by genome-scale metabolic modeling (GSMM), a computational platform that has allowed highly accurate predictions of which metabolic genes are essential in key organisms. GSMM uses the network structure of metabolism to predict insults that will harm biomass production. As such, it is a reliable engine for predicting essential genes, in addition to other related phenotypes such as reaction and metabolite essentiality and network robustness [4,8,9].

Here, we use a combination of GSMM and bioinformatics-level analysis to study the robustness of essential genes. In order to generalize our results beyond a single model organism, we perform our analyses both in *E*. *coli* and in yeast. Importantly, the analyses in each organism are done independently and do not make assertions or assumptions about which genes are essential across both *E*. *coli* and yeast, but rather are based on experimentally (or model-) derived essential gene sets in the relevant organism only.

In both *E*. *coli* and *S*. *cerevisiae*, we show that essential genes contain stronger genetic protections against mutations than non-essential genes, as they have higher scores in novel measures we term “coding efficiency” and “coding robustness”. These measures denote respectively a gene’s efficiency of translation and robustness to non-synonymous mutations (see Results Section: Sequence coding of essential genes is efficient and robust). We also show evidence that this model of vulnerability at one level being compensated by robustness at a lower level is a general property of biological networks, at least with regard to cell metabolism: we find that essential reactions are encoded by more robust genes than non-essential reactions, and that essential metabolites are produced via more reactions than non-essential metabolites. The results are true both for *Escherichia coli* and *Saccharomyces cerevisiae*, model prokaryote and eukaryote, respectively. Furthermore, we show that coding efficiency and coding robustness correlate positively with gene conservation level regardless of the essentiality of the genes. This indicates that true gene ‘essentiality’ might be best defined in a way that includes not only genetic redundancy, but the back-up mechanisms of coding efficiency and coding robustness as well.

## Results

### The different layers of cellular robustness

Living organisms have evolved to be highly robust. This is especially true among organisms that live in complex and hostile environments, which includes most human pathogens and biotechnologically relevant microbes. Considering the striking robustness of these organisms, we hypothesize that robustness mechanisms have formed to protect against the loss of essential genes, which lack the key property of redundancy. The question remains: if we are able to find such robustness mechanisms, what do they look like?

**Fig 1:**
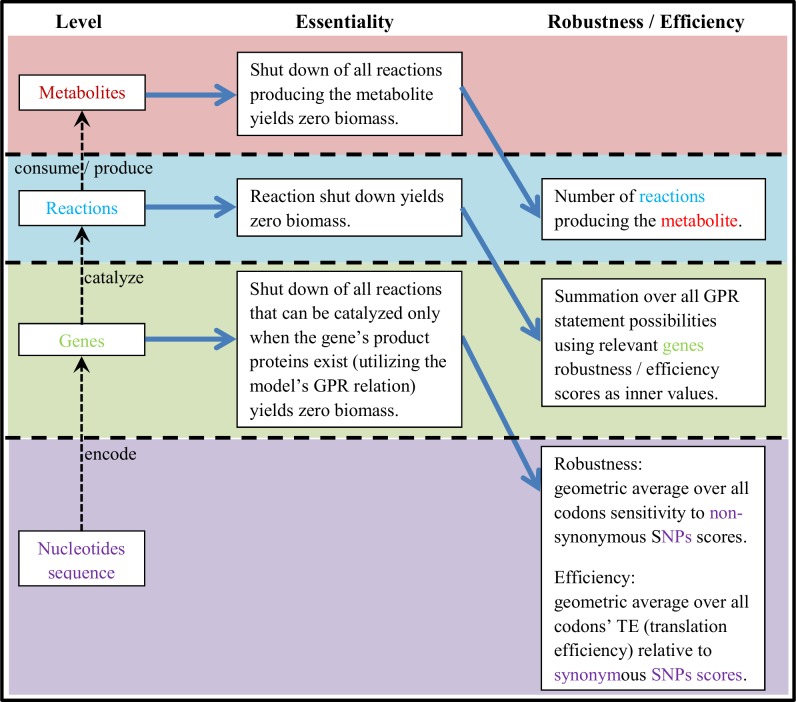
A four-level robustness hierarchy of the cell. Consecutive layers are separated by a horizontal dashed line. As evident, each element’s efficiency/robustness arises from components at the layer beneath it; metabolite production depends on the functioning of the reactions producing it, the latter depends on the function of the genes encoding their enzymes, and the genes’ sequence effects their robustness and efficacy in turn.

We hypothesize that the answer lies in the interconnected biochemical networks (e.g., metabolic, regulatory) that drive cellular function, which may employ varying levels of physical compensatory systems to render essential genes less vulnerable, and thus relieve the evolutionary pressure to back them up with alternative genes. To investigate this, we examined *E*. *coli* and *S*. *cerevisiae* at multiple scales, focusing on metabolic genes and the various systems in which they participate. Fig 1 shows the general scheme of our investigation. We find, indeed, that multiple levels of compensation exist in the cell, which uphold the general observation of cellular robustness as it relates to essential genes, and sheds light on how essential genes can persist across the tree of life without devastating consequences.

### Sequence coding of essential genes is efficient and robust

We began our study at the level of DNA, asking whether essential genes are preferentially coded to help protect the loss of their function. To test this, we considered the relative efficiency of translation of essential versus non-essential genes, using a standard measure of how good an organism’s ribosomes are at translating each codon (Methods). We examined this property, which we term “*coding efficiency*”, in several datasets of essential genes in *E*. *coli* identified under various growth conditions, as well as in *S*. *cerevisiae* under rich glucose medium. We found that, from a translation efficiency standpoint, essential genes are coded highly significantly more efficiently than non-essential genes (p-values < = 5.1e-9 in all essential gene sets available for *E*. *coli* or yeast, Wilcoxon one sided test; see Table 1). The same analysis on metabolic genes also yielded significant results (p-value < = 3.6e-4 for all essential gene sets tested; see Table 1).

**Table 1:** Coding efficiency of experimentally determined essential vs. non-essential genes.

Organism	Data set	Coding efficiency	
		All genes	Metabolic genes
*E*. *coli*	Gerdes et al., 2003 [10]	5.1e-9	7.7e-4
	Hashimoto et al., 2005 [11]	7.6e-51	3.3e-8
	Joyce et al., 2006 [12]	1.2e-18	7.3e-9
	Baba et al., 2006 [13]	2e-39	8.2e-7
*S*. *cerevisiae*	Giaever et al., 2002 [14]	8.6e-24	3.6e-4

P-values are reported, denoting the difference in coding efficiency between non-essential and essential genes in one-sided ranksum tests (checking for higher coding efficiency of essential genes). This analysis is done for all genes (left), as well as only including metabolic genes (right). The criterion for including a gene as ‘metabolic’ was that it exists in the *E*. *coli* or *S*. *cerevisiae* metabolic models.

It is possible that the differences we observe in coding efficiency between essential and non-essential genes are due simply to essential genes having generally higher expression levels, as it is known that expression level and coding efficiency are not independent [15–17]. To control for this, we redid our analysis of coding efficiency comparing genes only within similar expression levels. We did this using transcriptomics [18] and gene essentiality data [12] under glycerol-supplemented minimal media for *E*. *coli*, and transcriptomics [19] and essentiality [14] data measured in a rich (YPD) medium for yeast. Indeed, despite essential genes being generally more expressed than non-essential genes (p-value < 4.9e-21 for *E*. *coli*, p-value < 0.0017 for *S*. *cerevisiae* in 15 out of 18 conditions tested), we found significantly higher coding efficiency of essential vs. non-essential genes even when comparing only within similar expression levels (this held true for all expression levels except for those that contained too few genes for a reliable test–see S1 Fig).

Since coding efficiency improves translation speed [20–22], our results suggest that the coding of essential genes has evolved to speed up translation upon demand. We were curious if the coding of essential genes has also preferentially evolved to guard against translation errors. To examine this question, we developed a second new metric, termed “*coding robustness*,” which is the ability of a protein to maintain its functionality given random non-synonymous SNPs in the gene that codes it (Methods). We found that essential genes in both *E*. *coli* and *S*. *cerevisiae* are significantly more robust at the codon level than non-essential genes (p-values < 0.0025 when considering all genes, and p-value < 0.0234 for only metabolic genes; see Table 2).

**Table 2:** Coding robustness of experimentally determined essential vs. non-essential genes.

Organism	Data set	Coding robustness	
		All genes	Metabolic genes
*E*. *coli*	Gerdes et al., 2003 [10]	9.6e-5	0.0039
	Hashimoto et al., 2005 [11]	2.8e-6	5.8e-4
	Joyce et al., 2006 [12]	0.0025	9.5e-6
	Baba et al., 2006 [13]	0.0016	0.0158
*S*. *cerevisiae*	Giaever et al., 2002 [14]	1.3e-33	0.0234

Results are shown for all genes and for only essential vs. non-essential metabolic genes (metabolic genes determined according to *E*. *coli* and *S*. *cerevisiae* models).

**Fig 2:**
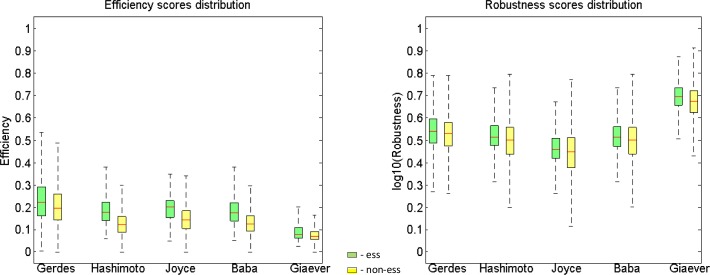
Distributions of coding efficiency and coding robustness scores of experimentaly determined essential and non-essential genes, Y-scale is normalized efficiency and normalized log10(robustness) values. Essential genes are significantly more efficient/robust in all cases, as described above (see Table 1 and Table 2).

This supports the notion that the potential detrimental implications of essential genes loss are mitigated by multiple mechanisms within cells. Fig 2 shows the essential and non-essential gene distributions of coding efficiency and coding robustness scores over all experiments described above–all of these distributions show significant differences between essential and non-essential gene groups.

Importantly, we never compare coding efficiency or coding robustness scores directly across *E*. *coli* and yeast, as these scores could have a different scaling in different organisms. Rather, all analyses with these metrics are done between genes with one organism only.

### Gene evolutionary conservation analysis supports the role of coding efficiency in protecting evolutionarily important, conserved genes

Evolutionary conservation is a key metric for testing what is truly important for a cell. For example, if modifications in essential genes were a major liability for cell survival, we would expect to observe higher selective pressure on them than on non-essential genes. Such a division should show a distinct and measurable signature in each gene’s evolutionary rate. A conventional metric for the latter is the dN/dS value, which tests the ratio of non-synonymous to synonymous substitutions seen in each gene within a family of organisms. Past studies are unclear about whether essential genes are more conserved than non-essential genes, showing at best weak but significant relationships [7,23,24]. Therefore, we checked this feature first, using dN/dS values for *E*. *coli* and yeast from [25,26]. We found that, in four out of five tested datasets, essential genes are significantly more conserved than non-essential genes. A similar test focusing only on metabolic genes showed significant conservation of essential vs. non-essential genes in yeast (but not in *E*. *coli*–see Table 3).

**Table 3:** Conservation p-values for 1-sided Ranksum tests generated from experiments and genome scale metabolic models.

Organism	Data set	p-value
*E*. *coli*	Gerdes et al., 2003 [10]	0.055
	Hashimoto et al., 2005 [11]	0.0098
	Joyce et al., 2006 [12]	0.0426
	Baba et al., 2006 [13]	0.0331
	GSM (metabolic genes)	0.8824
*S*. *cerevisiae*	Giaever et al., 2002 [14]	6.09e-30
	GSM (metabolic genes)	0.0159

**Fig 3:**
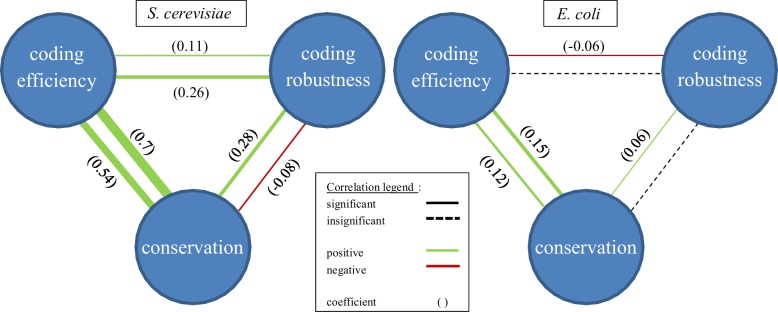
Correlation triangle scheme between coding efficiency, coding robustness and evolutionary conservation scores, for *S*. *cerevisiae* and *E*. *coli*. Outer lines state genome wide correlations while inner lines state correlations of metabolic genes. Edges thickness represents the correlation coefficient.

The finding of high conservation among essential genes confirms the importance to cells of evolving other compensatory mechanisms. To gain a better understanding of how conservation and robustness relate, we analyzed the correlation between the conservation data and coding efficiency and coding robustness scores of all genes and metabolic genes (Fig 3; also, see S1 Table for details). This analysis reveals that evolutionary conservation levels and coding efficiency levels of genes, and to a lesser extent, also coding robustness for metabolic genes, are strongly associated in yeast (Fig 3). Although the correlation values are much weaker in *E*. *coli*, a Ranksum-based analysis still shows significant associations (S2 Fig). These results point to the key role of coding efficiency in protecting evolutionary important, conserved genes, although it remains a puzzle why the association of conservation with robustness measures is so much weaker in *E*. *coli*.

We next analyzed coding efficiency versus coding robustness in *E*. *coli* and yeast, to see if their levels correlate across genes (top of triangles in Fig 3). We found that coding efficiency and coding robustness levels correlate only weakly in yeast, and non-significantly in *E*. *coli*. Since robustness and efficiency are separate and in some ways complementary mechanisms, we didn’t necessarily expect to see a correlation between them–efficiency, robustness, or both could be at play in a given organism, leading to different compensational strategies in different species.

### Like genes, metabolites and metabolic reactions are backed-up at other levels of the metabolic network

Our emerging hypothesis is that essential genes contain several compensatory mechanisms that render their essentiality less dangerous to cell survival, in accordance with the general observation that, like all evolved stable systems, cells are robust. We next asked if this concept can be generalized to other cellular elements, which fill higher levels in the cell’s functional hierarchy. For this we focused on metabolism, since its network structure is the best known and it exhibits clear modes of essentiality that are more readily amenable to a computational analysis than other cellular processes such as, e.g., regulation and signaling.

To this end we used genome-scale metabolic models (GSM), which have been built for both *E*. *coli* [27] and *S*. *cerevisiae* [28], and provide a solid platform for analysis of a variety of metabolic features. An advantage of GSM analysis is that we can examine network features in a broad range of *in silico* conditions, thereby exposing our models to a much broader range of conditions than might be possible in lab experiments. For the analyses we describe here, we assessed the models in 2000 random viable growth media, thus spanning a large space of potential *in silico* environments and thus ensuring broadly defined concepts of essentiality and robustness (see Methods).

**Fig 4:**
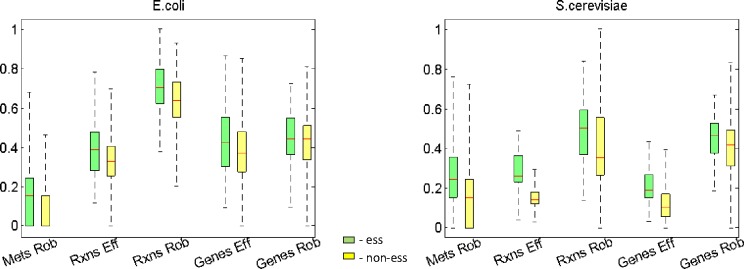
Distributions of efficiency and robustness scores of essential vs non-essential elements generated from genome scale models of *E*. *coli* and *S*. *cerevisiae*. Y-scale is normalized efficiency and normalized log10(robustness) values. Mets–metabolites, Rxns–reactions, Eff–efficiency, Rob–robustness.

First, we identified two types of key metabolic elements to assess: metabolic reactions and the metabolites themselves. We determined the essential members of each set by assessing which ones, when removed from the metabolic network, eliminate *in silico* biomass production. Next, we defined robustness and efficiency scores for each element as appropriate, analogous to the scores we described for essential genes. Namely, we derived the efficiency and robustness of a reaction from the coding efficiency and coding robustness of the underlying genes, as calculated using gene-protein-reaction Boolean logic (see Methods). We determined the robustness of a metabolite as the number of reactions in the metabolic network that are able to produce it (i.e., the redundancy built into the system). We found that essential elements overwhelmingly exhibit more efficiency and robustness than non-essential elements, with 9 of 10 comparisons yielding significance in Ranksum tests (Table 4). Fig 4 shows the essential and non-essential elements distributions of efficiency and robustness scores. This considerably extends our essential gene backup hypothesis to multiple cell layers.

**Table 4:** Efficiency and robustness 1-sided Ranksum p-values generated from genome scale metabolic models.

Model	Metabolites robustness	Reactions		Genes	
		Efficiency	Robustness	Efficiency	Robustness
*E*. *coli*	1.29e-6	0.0058	2e-4	0.0108	0.0771
*S*. *cerevisiae*	8.45e-7	3.61e-12	0.0117	3.5e-6	0.022

### Acetylated proteins are enriched for essential genes

Following our overall hypothesis about the existence of backup mechanisms, we performed a preliminary analysis to determine if post-translational modifications also might play a role in backing up essential genes. Specifically, we studied the post-translational modification of lysine acetylation, which plays a key role in regulating chromatin dynamics, gene expression, and metabolic pathways both in eukaryotes and in prokaryotes [29–32]. When analyzing a dataset of acetylated proteins in *E*. *coli* [33], we found that they are highly enriched with essential genes (S2 Table). That is, *E*. *coli* proteins encoded by essential genes are more prone to undergo this type of post-translational modification than proteins encoded by non-essential genes. The extent to which such acetylation modifications confer robustness is yet an open question.

## Discussion

Our launching off point in this study is the question: what robustness mechanisms are in place to protect cells against the loss of essential genes, which otherwise might represent significant sources of vulnerability in single-celled organisms? We start by defining two complementary gene features, *coding efficiency* and *coding robustness*, and showing that essential genes tend to be encoded more efficiently and robustly than non-essential genes in both *E*. *coli* and yeast. This result supports a previously observed relation between structurally and functionally more important sites and high translation efficiency [34], and importantly, extends it to the gene level. We next show that the compensatory mechanisms that protect against essential gene loss, including and especially the mechanism of coding efficiency, are positively associated with the genes’ evolutionary conservation rates. This testifies to the functional importance of these properties.

Unlike the other robustness and efficiency metrics we explored, *E*. *coli* genes’ coding robustness was not found to associate significantly more strongly with essential genes than non-essential ones when essentiality was determined in context of the ability of a metabolic model to produce biomass (see Table 4). While this result is surprising, it is possible that it is an artifact due to mis-labeling of the essentiality of some genes, especially since the result conflicts with that seen with experimentally validated essential gene sets (compare Table 2 with the rightmost column of Table 4). This could easily happen if, for example, a gene is essential under evolutionarily relevant conditions, but is marked non-essential because there are environments (albeit non-evolutionarily relevant ones) in which the metabolic model predicts it is not necessary for cell survival.

Beyond our main analyses of coding robustness and coding efficiency, we also explored two generalizations of our main hypothesis. First, we explored whether back-up mechanisms exist for essential elements other than genes–namely, essential metabolic reactions and essential metabolites. Indeed, we found that essential metabolic reactions are encoded by more “robust” genes than non-essential reactions, and that essential metabolites are produced by more reactions than non-essential metabolites. This generalizes our initial result, showing that essential elements in metabolic networks also display compensatory mechanisms.

Our second generalization of the main hypothesis involved checking if there might also be mechanisms at the post-translational level that protect the loss of essential genes. While our analysis is preliminary and only correlative, we found that essential genes are enriched among acetylated proteins, suggesting that this may constitute another method of back-up. Fully vetting this hypothesis is out of the scope of this study, but when taken alongside our finding of backup mechanisms for essential metabolites and essential reactions in metabolism, it suggests the possible existence of yet other backup mechanisms for essential genes (and other essential cellular elements), which we have not yet identified, but that are yet present and active in cells.

If such mechanisms exist, this might explain one puzzling result that we found in our analysis, that coding robustness and coding efficiency were both more strongly conserved among metabolic genes than non-metabolic genes (in both yeast and *E*. *coli—*see Fig 3). Namely, it would suggest that there might be a range of other, as-yet-unidentified compensational mechanisms that play a stronger role in non-metabolic processes than metabolic ones, thus alleviating the need for robust or efficient genes to be conserved in non-metabolic pathways. Identifying each of these specific features and understanding the elaborate synergies between them may give an entirely new perspective on network essentiality, and on how it affects cellular fitness as a whole.

## Materials and Methods

### Genome Scale Metabolic Modeling (GSMM)

A metabolic network consisting of *m* metabolites and *n* reactions can be represented by a *stoichiometric matrix S*, where the entry *S*_*ij*_ represents the stoichiometric coefficient of metabolite *i* in reaction *j* [35]. A CBM model imposes mass balance, directionality and flux capacity constraints on the space of possible fluxes in the metabolic network's reactions through a set of linear equations

S*v=0 (1)

$$v_{min}\leq v\leq v_{max}$$ (2)

Where *v* stands for the flux vector for all of the reactions in the model (i.e. the *flux distribution*). The exchange of metabolites with the environment is represented as a set of *exchange (transport) reactions*, enabling a pre-defined set of metabolites to be either taken up or secreted from the growth media. The steady-state assumption represented in Eq (1) constrains the production rate of each metabolite to be equal to its consumption rate. Enzymatic directionality and flux capacity constraints define lower and upper bounds on the fluxes and are embedded in Eq (2). In the following, flux vectors satisfying these conditions will be referred to as feasible steady-state flux distributions. Gene knock-outs are simulated by constraining the flux through the corresponding metabolic reaction to zero.

### Essentiality in the metabolic model

In order to determine essentiality values, each element (gene/reaction/metabolite) was KOed in the following way: *Gene KO*–block the flux of all reactions with GPR statement that always returns ‘false’ when the value of the current gene relevant variable is set to ‘false’ (by changing the upper and lower limits to zero); *Reaction KO*–block the flux of the specific reaction; *Metabolite KO*–block the flux of every reaction that consumes this metabolite (and make sure there is an appropriate exchange reaction, to preserve the steady-state constraint). According to the effect of the KO on the maximal biomass production, one of three essentiality values was assigned to each element: *Essential*–KO reduces biomass production to zero; *Non-essential*–KO doesn’t affect the biomass at all (compared to the wild type); *Undefined*–KO reduces biomass production but not completely, these elements (which constitute a small minority) are ignored in the analysis.

### Genes’ coding efficiency

Synonymous mutations do not alter the produced protein, yet can have a significant effect on protein levels in the cell [36]. This score measures relative translation efficiency levels of genes, achieved by preferring some codons over (synonymous) others. To quantify the relative translational efficiency, we use the tRNA Adaptation Index (tAI) [37], as also described in [38]. Briefly, each codon is assigned with a TE value based on the availability of the corresponding tRNAs in the cell (as reflected by their genomic copy numbers). A codon score is achieved by multiplying all ratios between its TE value and the TE values of all synonymous codons that can be achieved by a SNP (see example below). A gene score is achieved by a geometric average over all of its codons’ scores. Our method is somewhat similar to using the RSCU metric, but the two are not the same. RSCU also measures the tendency of preferring some codons over others, although not necessarily more efficiently translated ones. Different environmental conditions can cause a bias towards codons that are less efficiently translated (but more adapted to the specific environment), thus having a high RSCU score [17]. Also, higher RSCU values can result from several reasons, such as mutational bias [39], and gene expression. In our work we wanted to analyze the effect of selection for translational efficiency on the codon bias and that is why we used the described method. Gene sequences for *S*. *cerevisiae* and *E*. *coli* were taken from KEGG [40] and BioMart [41], respectively. Codons TE and efficiency scores are presented in S3 Table.

Codons efficiency score calculation example:

$${EFFICIENCY}_{CCG}=({TE}_{CCG}/{TE}_{CCT})*({TE}_{CCG}/{TE}_{CCC})*({TE}_{CCG}/{TE}_{CCA})=(0.165/0.054875)*(0.165/0.125)*(0.165/0.125)=3.007*1.32*1.32=5.239107$$

### Genes’ coding robustness

As opposed to synonymous SNPs, non-synonymous SNPs change the produced protein and therefore can disrupt or even totally disable it. Each protein functionality level is derived from its amino acids chain, and the robustness metric we define here aims to measure the functionality degradation caused by non-synonymous SNPs. We assume that common variants of the protein are a result of undamaging / minor damaging mutations. By utilizing that assumption, we use the *amino acid substitution matrix*, PAM70 [42], to assign a score to all codons. This is done by multiplying all relevant entries in the PAM70 matrix, representing all possible resulting codons due to a non-synonymous SNP (see example below). Here also, a gene score is achieved by a geometric average over all of its codons’ scores. Because the score is calculated independently over all of the codons in the sequence, our method doesn’t take into account SNPs that result in a stop codon (which will turn the rest of the sequence invalid). Codons robustness scores are presented in S3 Table.

Codons robustness score calculation example:

$${ROBUSTNESS}_{GCT}={AA}_{\left[GCT,TCT\right]}*{AA}_{\left[GCT,CCT\right]}*{AA}_{\left[GCT,ACT\right]}*{AA}_{\left[GCT,GTT\right]}*{AA}_{\left[GCT,GAT\right]}*{AA}_{\left[GCT,GGT\right]}=e^{1}*e^{0}*e^{1}*e^{-1}*e^{-1}*e^{0}=1$$

### Reactions’ efficiency and robustness

Each reaction in the metabolic model has its own boolean GPR (Gene Protein Relation) statement which determines if the reaction will be catalyzed according to expressed genes. An efficiency / robustness score is obtained by a summation over all possible entries in the induced truth table of the reaction’s GPR. Instead of using a simple boolean values (thus treating all genes as uniformly expressed), we used relevant genes efficiency / robustness scores (and min/max operations instead of and/or).

### Compiling essential network components from the metabolic model

We generated 2000 random viable media for each organism metabolic model, allowing the production of cellular biomass. Utilizing these media, we obtained 2000 different metabolic networks, which resulted in 2000 essential components groups in both *E*. *coli* and *S*. *cerevisiae*. To avoid bias due to dead-end elements in the model we considered only: live reactions, genes that appear in live reactions’ GPR and metabolites that are produced by at least one reaction. Also, in the metabolites case, we tested only those that do not take part in the biomass reaction, since we want to find metabolites that block the biomass reaction for a reason other than being part of the biomass function itself. An element was considered essential if it was essential over all random models. In order to validate the essential components groups, they were compared to an essentiality test results using rich media. An element was considered non-essential if its KO did not affect biomass production under all random models; Thus composing lists of elements with essentiality trait that is determined by the model itself and not affected by a given media. A convergence test was used to determine that the number of random media was sufficient. Lists of the always-essential and always-non-essential metabolic elements can be found in S1 and S2 Files, respectively.

## Supporting Information

S1 Fig Comparison between essntial and non essential genes with similar expresstion values. (PDF)

S2 Fig Wilcoxon one sided test of efficiency and robustness scores between genes with high vs. low conservation. (PDF)

S1 File *E*. *Coli* always-essential and always-non-essential metabolic elements. (XLSX)

S2 File *S*. *cerevisiae* always-essential and always-non-essential metabolic elements. (XLSX)

S1 Table Correlation between conservation, efficiency and robustness scores, for *E*. *coli* and *S*. *cerevisiae*. (PDF)

S2 Table Hypergeometric p-values, measuring the random overlap probability between genes that encode lysine acetylated proteins and essential genes from several *E*. *coli* datasets. (PDF)

S3 Table Codons’ efficiency and robustness scores. (PDF)
