# Supplementary material for: Essential Genes Embody Increased Mutational Robustness to Compensate for the Lack of Backup Genetic Redundancy
Source: PLoS One. 2016 Dec 20;11(12):e0168444. doi: 10.1371/journal.pone.0168444 (PMC5173180; doi:10.1371/journal.pone.0168444)

S1 Fig. Comparison between essntial and non essential genes with similar expresstion values.

Each x-axis value represents the middle of a (sliding) window with a percentile margine of 40% from which the relevant genes were compared. Y-axis values are $–\log_{10}$(p-value), where p-value is the result of a wilcoxon one sided test between the relevant essential and non-essential genes. Significance threshold ($–\log_{10}(0.05)$) is marked by a horizontal black line. Each colored curve corresponds to a different expression vector extracted from different sample in each dataset.

Using an expression range with a margin lower than 40% for the comparison only raised the minimal expression threshold for significance (because there are few essential genes with low expression levels).

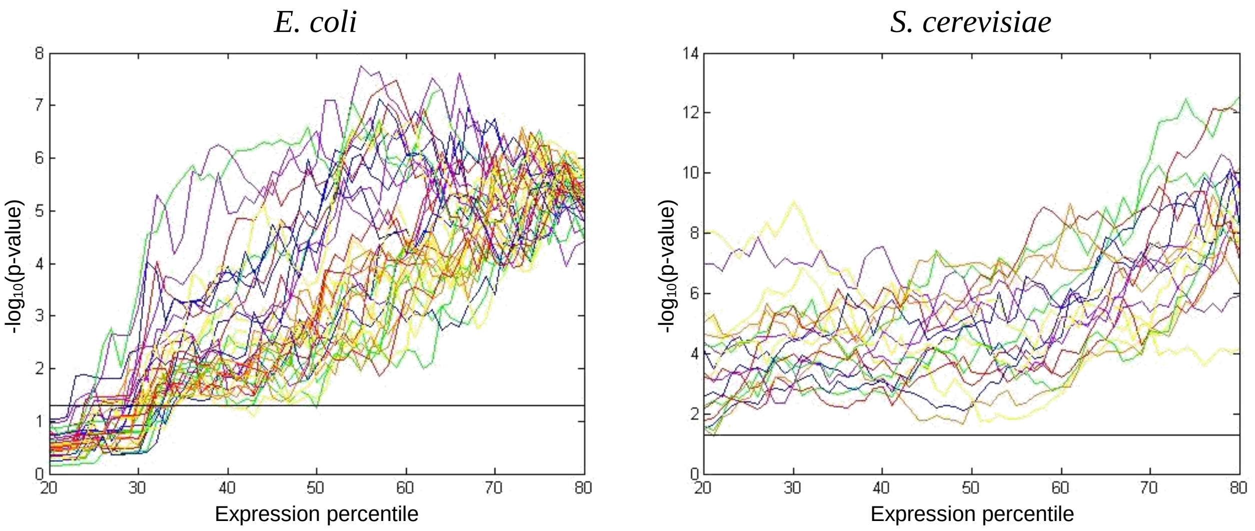

Supplement: S1 Fig — (PDF) [file pone.0168444.s001.pdf]
