# Supplementary material for: Essential Genes Embody Increased Mutational Robustness to Compensate for the Lack of Backup Genetic Redundancy
Source: PLoS One. 2016 Dec 20;11(12):e0168444. doi: 10.1371/journal.pone.0168444 (PMC5173180; doi:10.1371/journal.pone.0168444)
Supplement: S2 Fig — (PDF) [file pone.0168444.s002.pdf]

S2 Fig. Wilcoxon one sided test of efficiency and robustness scores between genes with high vs. low conservation.

X axis represents genes with conservation scores above 'x' percentile, which are compared against genes with conservation scores of below 100-x percentile. Blue lines mark the size of the compared gene groups. Brown and red lines mark the measured p-value for efficiency and robustness (respectively). Areas of significance are stated by a yellow background. a) Yeast – genome wide (since correlation between robustness and conservation here is negative, 1-robustness values were used). b) Yeast – metabolic genes. c) *E. coli* – genome wide. b) *E. coli* – metabolic genes.

a)

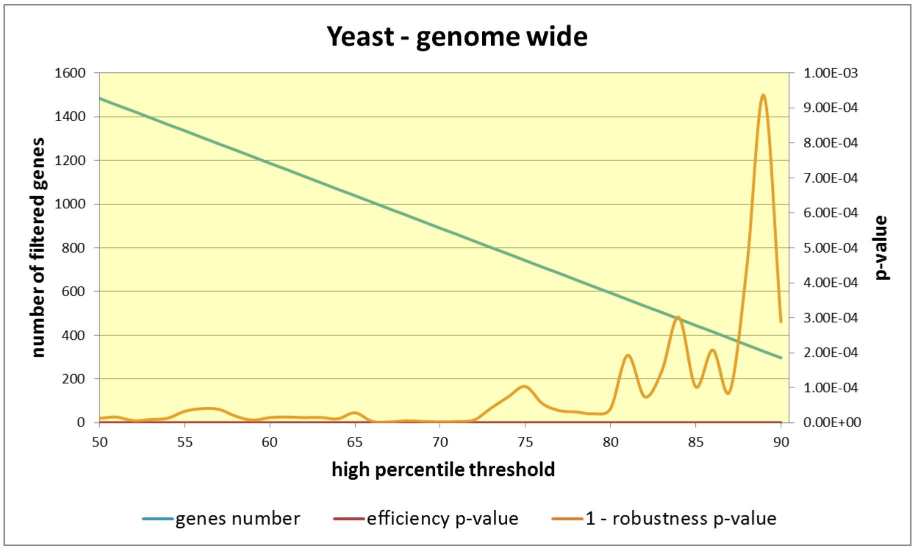

b)

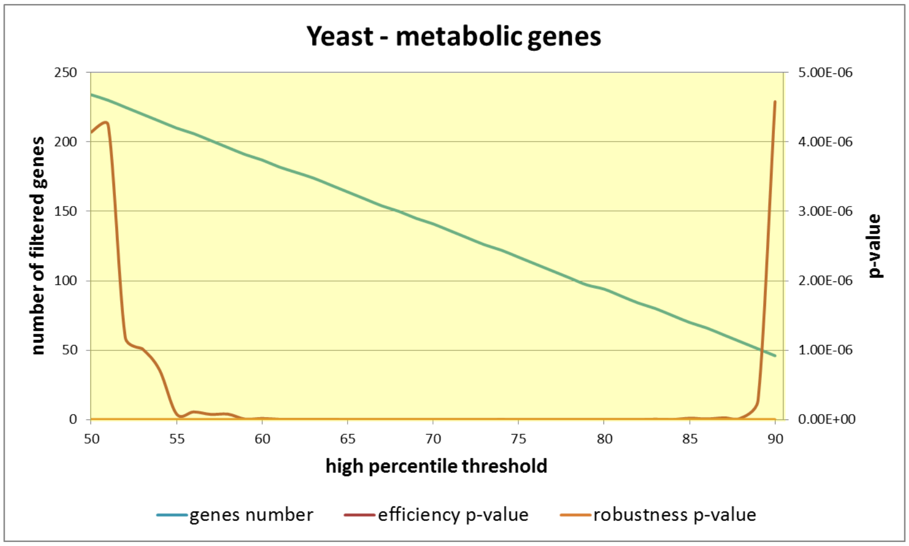

c)

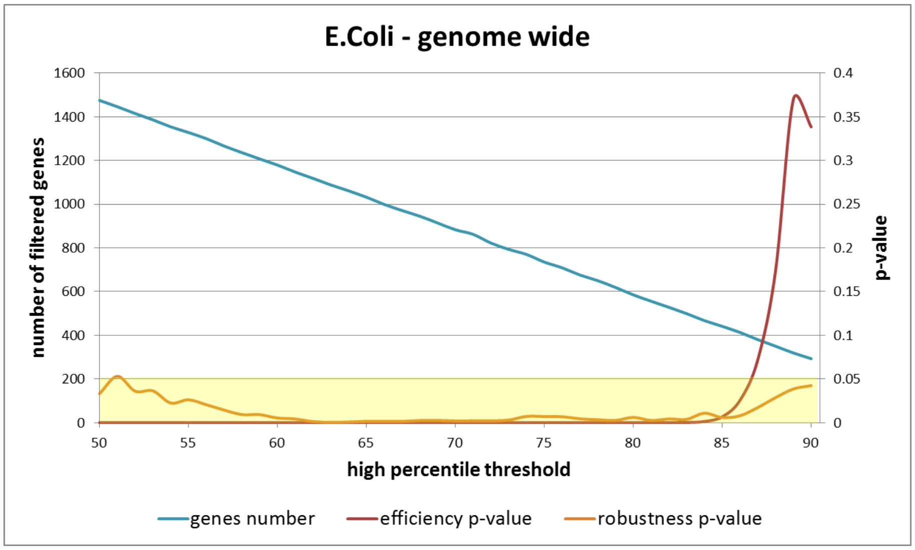
E.Coli - genome wide
number of filtered genes
p-value
1600
1400
1200
1000
800
600
400
200
0
0.4
0.35
0.3
0.25
0.2
0.15
0.1
0.05
0
50
55
60
65
70
75
80
85
90
high percentile threshold
genes number
efficiency p-value
robustness p-value

d)

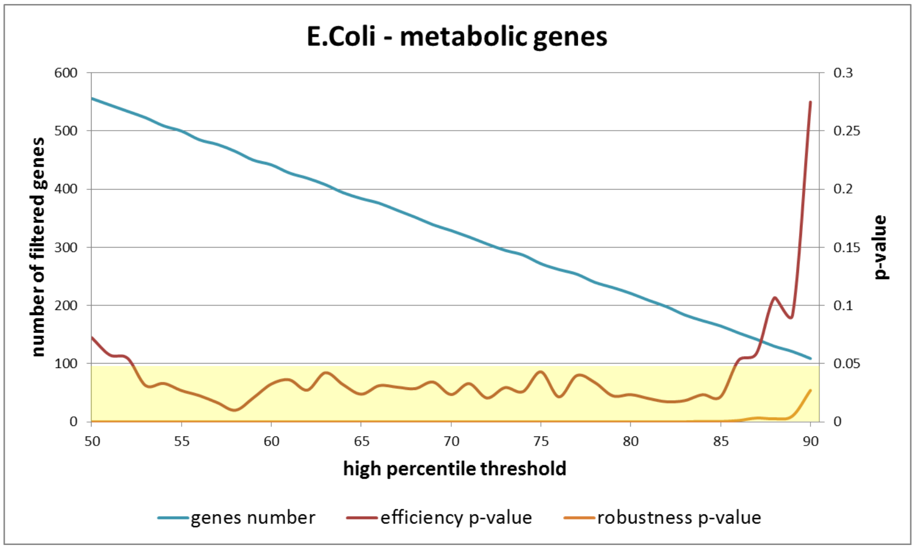
E.Coli - metabolic genes
number of filtered genes
p-value
600
500
400
300
200
100
0
0.3
0.25
0.2
0.15
0.1
0.05
0
50
55
60
65
70
75
80
85
90
high percentile threshold
genes number
efficiency p-value
robustness p-value
