# Supplementary material for: Essential Genes Embody Increased Mutational Robustness to Compensate for the Lack of Backup Genetic Redundancy
Source: PLoS One. 2016 Dec 20;11(12):e0168444. doi: 10.1371/journal.pone.0168444 (PMC5173180; doi:10.1371/journal.pone.0168444)
Supplement: S1 Table — (PDF) [file pone.0168444.s005.pdf]

S1 Table. Correlation between conservation, efficiency and robustness scores, for *E. coli* and *S. cerevisiae*.

**Organism**		**Correlation**	**Genes**	**coefficient**	**p-value**
E. coli	Genome wide	efficiency - robustness	4259	-0.0622	4.81e-05
		efficiency – conservation	2944	0.1231	2.03e-11
		robustness - conservation		0.0034	0.054
	model	efficiency - robustness	1304	-0.0245	0.3759
		efficiency – conservation	1115	0.1478	7.17e-7
		robustness - conservation		0.0611	0.0413
S. cerevisiae	Genome wide	efficiency - robustness	6043	0.1011	3.37e-15
		efficiency – conservation	2962	0.5441	6.75e-228
		robustness - conservation		-0.0824	7.14e-6
	model	efficiency - robustness	874	0.2588	7.63e-15
		efficiency – conservation	475	0.7025	0
		robustness - conservation		0.2835	3.15e-10
