# Supplementary material for: Essential Genes Embody Increased Mutational Robustness to Compensate for the Lack of Backup Genetic Redundancy
Source: PLoS One. 2016 Dec 20;11(12):e0168444. doi: 10.1371/journal.pone.0168444 (PMC5173180; doi:10.1371/journal.pone.0168444)
Supplement: S2 Table — (PDF) [file pone.0168444.s006.pdf]

S2 Table. Hypergeometric p-values, measuring the random overlap probability between genes that encode lysine acetylated proteins and essential genes from several *E. coli* datasets.

**Data set**	**HG p-value**
Gerdes et al., 2003 [10]	3.38e-12
Hashimoto et al, 2005 [11]	9.68e-35
Joyce et al., 2006 [12]	6.13e-05
Baba et al., 2006 [13]	7.91e-26
GSM	2.61e-37
