# Supplementary material for: Essential Genes Embody Increased Mutational Robustness to Compensate for the Lack of Backup Genetic Redundancy
Source: PLoS One. 2016 Dec 20;11(12):e0168444. doi: 10.1371/journal.pone.0168444 (PMC5173180; doi:10.1371/journal.pone.0168444)
Supplement: S3 Table — (PDF) [file pone.0168444.s007.pdf]

S3 Table. Codons' efficiency and robustness scores.

Codon	amino acid	*E. coli*		*S. cerevisiae*		robustness
		TE	efficiency	TE	efficiency	
TTT	Phe	0.10975	0.439	0.27032	0.438999	1.13E-07
TTC	Phe	0.25	2.277904	0.615764	2.277908	1.13E-07
TTA	Leu	0.125	0.757576	0.431034	1.334419	0.0009119
TTG	Leu	0.165	0.403333	0.753695	22.29445	4.54E-05
TCT	Ser	0.10975	0.256377	0.67734	28.57028	0.0009119
TCC	Ser	0.25	6.90274	0.487685	7.67795	0.0009119
TCA	Ser	0.125	0.431421	0.184797	0.158295	0.0497871
TCG	Ser	0.165	1.309777	0.12069	0.028799	0.0024788
TAT	Tyr	0.164625	0.439	0.216256	0.439	8.32E-07
TAC	Tyr	0.375	2.277904	0.492611	2.277907	8.32E-07
TAA	Stop	-1	1	-1	1	1
TAG	Stop	-1	1	-1	1	1
TGT	Cys	0.054875	0.439	0.108128	0.439	1.71E-15
TGC	Cys	0.125	2.277904	0.246305	2.277902	1.71E-15
TGA	Stop	-1	1	-1	1	1
TGG	Trp	0.165	1	0.369458	1	1.15E-17
CTT	Leu	0.054875	0.019584	0.027032	0.029377	3.06E-07
CTC	Leu	0.125	0.527293	0.061576	0.79093	3.06E-07
CTA	Leu	0.125	0.527293	0.184729	27.45719	2.26E-06
CTG	Leu	0.54	601.0305	0.059113	0.052688	6.14E-06
CCT	Pro	0.054875	0.064094	0.123153	0.173609	1.67E-05
CCC	Pro	0.125	1.725685	0.08867	0.046655	1.67E-05
CCA	Pro	0.125	1.725685	0.615776	108.5134	4.54E-05
CCG	Pro	0.165	5.239107	0.197044	1.137746	4.54E-05
CAT	His	0.054875	0.439	0.189224	0.439	0.0497871
CAC	His	0.125	2.277904	0.431034	2.277903	0.0497871
CAA	Gln	0.25	0.757576	0.554187	2.319589	2.7182818
CAG	Gln	0.33	1.32	0.238916	0.431111	2.7182818
CGT	Arg	0.5	55555.56	0.369458	83211.64	2.06E-09
CGC	Arg	0.36	14929.92	0.26601	22362.26	2.06E-09
CGA	Arg	0.00005	2.22E-15	0.000037	4.57E-16	8.32E-07
CGG	Arg	0.125	164.4045	0.061576	14.2046	8.32E-07
ATT	Ile	0.164625	0.442822	0.800493	9.0219	0.0497871
ATC	Ile	0.375	5.234027	0.576355	3.367407	0.0497871
ATA	Ile	0.163204	0.431454	0.123233	0.032916	0.1353353
ATG	Met	1	1	0.615764	1	20.085537

Codon	amino acid	*E. coli*		*S. cerevisiae*		robustness
		TE	efficiency	TE	efficiency	
ACT	Thr	0.10975	0.14587	0.67734	18.4221	7.3890561
ACC	Thr	0.25	3.927421	0.487685	4.950736	7.3890561
ACA	Thr	0.125	0.245464	0.246373	0.322467	0.0067379
ACG	Thr	0.29	7.111144	0.140394	0.034002	0.0024788
AAT	Asn	0.2195	0.439	0.27032	0.438999	0.3678794
AAC	Asn	0.5	2.277904	0.615764	2.277908	0.3678794
AAA	Lys	0.75	3.125	0.431034	0.431034	0.0024788
AAG	Lys	0.24	0.32	1	2.320003	0.1353353
AGT	Ser	0.054875	0.439	0.054064	0.438999	0.0067379
AGC	Ser	0.125	2.277904	0.123153	2.277911	0.0067379
AGA	Arg	0.125	1893.939	0.67734	44551.21	2.26E-06
AGG	Arg	0.165	1.7424	0.278325	1.857318	6.14E-06
GTT	Val	0.10975	0.042302	0.862069	51.52098	1.67E-05
GTC	Val	0.25	1.138952	0.62069	13.8457	1.67E-05
GTA	Val	0.625	44.49032	0.123239	0.021518	0.0067379
GTG	Val	0.2	0.466515	0.162562	0.065147	0.0003355
GCT	Ala	0.10975	0.117506	0.67734	21.00232	1
GCC	Ala	0.25	3.163756	0.487685	5.644143	1
GCA	Ala	0.375	16.01651	0.30795	0.897349	1
GCG	Ala	0.12	0.167945	0.098522	0.009401	1
GAT	Asp	0.164625	0.439	0.432512	0.439	0.0024788
GAC	Asp	0.375	2.277904	0.985222	2.277907	0.0024788
GAA	Glu	0.5	3.125	0.862069	2.160493	0.3678794
GAG	Glu	0.16	0.32	0.399015	0.462857	0.3678794
GGT	Gly	0.2195	1.025509	0.432512	2.439044	1.13E-07
GGC	Gly	0.5	27.61096	0.985222	65.66945	1.13E-07
GGA	Gly	0.125	0.107855	0.184729	0.081165	4.14E-08
GGG	Gly	0.165	0.327444	0.182266	0.076922	1.88E-12
